# Dynamic Resource Allocation in Disaster Response: Tradeoffs in Wildfire Suppression

**DOI:** 10.1371/journal.pone.0033285

**Published:** 2012-04-13

**Authors:** Nada Petrovic, David L. Alderson, Jean M. Carlson

**Affiliations:** 1 Center for Research on Environmental Decisions, Columbia University, New York, New York, United States of America; 2 Operations Research Department, Naval Postgraduate School, Monterey, California, United States of America; 3 Physics Department, University of California, Santa Barbara, Santa Barbara, California, United States of America; University of Namur, Belgium

## Abstract

Challenges associated with the allocation of limited resources to mitigate the impact of natural disasters inspire fundamentally new theoretical questions for dynamic decision making in coupled human and natural systems. Wildfires are one of several types of disaster phenomena, including oil spills and disease epidemics, where (1) the disaster evolves on the same timescale as the response effort, and (2) delays in response can lead to increased disaster severity and thus greater demand for resources. We introduce a minimal stochastic process to represent wildfire progression that nonetheless accurately captures the heavy tailed statistical distribution of fire sizes observed in nature. We then couple this model for fire spread to a series of response models that isolate fundamental tradeoffs both in the strength and timing of response and also in division of limited resources across multiple competing suppression efforts. Using this framework, we compute optimal strategies for decision making scenarios that arise in fire response policy.

## Introduction

Wildfire progression is driven by inherently complex, interconnected, physical processes, involving a variety of factors, including weather, vegetation, and terrain [1]–[4]. Wildfires are difficult to predict in space and time, and thus it is hard to make informed decisions about mitigation efforts, particularly the allocation of limited resources during dynamic response. Furthermore, the areas and damages of wildfires both exhibit high variability, heavy tailed statistics [5], implying that overall impacts are dominated not by the typical, median size events, but rather by the relatively rare, large events. This highlights the fundamental importance of understanding tradeoffs between strength and timing in mitigation efforts that may help prevent future wildfires from growing out of control.

In recent years, wildfires in the United States have received media attention due to rising suppression costs [6], which have regularly exceeded expenditures of a billion dollars per year in the new millennium [7]. A variety of potential driving factors have been suggested [8], including a climate-driven increase in wildfire activity [9]–[11], and development at the wildland urban interface [12], [13]. A cohesive strategy that takes into account both the natural occurrence of wildfire on the landscape and its impact on human lives is needed to determine the best course of action for reducing these costs. The Federal Land Assistance, Management and Enhancement Act (FLAME Act) of 2010 directs the U.S. Department of Agriculture, the U.S. Department of the Interior, and the U.S. Department of Homeland Security to develop a Cohesive Wildfire Management Strategy to identify, among other things, the most cost effective means for allocating fire management budget resources. Thus, there is a need to understand not only the dynamics underlying wildfire behavior but also how to allocate limited resources to mitigate fire damage.

In this paper we introduce a minimal model of wildfire dynamics using the formalism of stochastic processes and queueing theory. We divide the burnable substrate, which may be forest, shrubland, or grassland depending on the region, into discrete parcels that arrive in a queue when they begin to burn. The growth rate of the number of parcels in the queue scales with the current size of the queue, capturing the escalating dynamics familiar in wildfire spread. Parcels exit the queue when they are extinguished either by natural causes or human suppression. This model reproduces the observed statistical distribution of wildfire sizes, and it enables investigation of tradeoffs in suppression efforts that are fundamental to decision making.

Our model is sufficiently general that it may apply to other scenarios involving coupled dynamics of a disaster and response. In the context of wildfires, our study is intended to be an intermediate step between models with static fires [14], [15] and models with a detailed fire spread simulation [16], [17]. It is not intended to replace high fidelity simulations but will be used to examine basic relationships, which can then be further explored and verified with more realistic models. We comment on this in more detail in the Discussion.

We focus specifically on time dynamics. However, spatial factors such as prepositioning of resources are still implicitly included through the time delay of resource arrival. Tradeoffs in strength and timing are particularly relevant when considering resource allocations to simultaneously occurring fires, which may be evolving on different time scales.

### Our Contributions in Context

There are many research efforts that are complementary to ours, having focused primarily on spatial rather than temporal aspects of the problem. A rich literature exists within operations research, focusing on optimization of wildfire response [18], including models with realistic fire spread and various types of suppression resources [16], [17], [19]. Because of the computational complexity inherent in simulating a single fire, much less multiple co-occurring fires or a statistical distribution of fires over time, these models often abstract away the time dynamics of individual fires. In many existing studies, the decision variable relates to spatial prepositioning of resources for the initial response. However, because wildfires are fundamentally both geospatial and dynamic, decision making tools must ultimately capture both the spatio-temporal spread of the fire itself and the geographic locations and transportation of suppression resources.

One aspect of wildfires that has received considerable attention in the complex systems literature is the power law distribution of fire sizes: 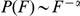 where *F* is the footprint (i.e., total burn area) of the fire and 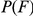 is the probability of a fire being larger than or equal to size *F*. Fires are not unique in this regard; many disparate disaster phenomena exhibit power law statistics when losses are tallied [20], [21], and many biological, technological, and social systems also exhibit power laws [22]–[24]. Although these observations have led to widespread association of power law statistics with a key signature of self-organization in complex systems [25], [26], there are mathematical, statistical, and data-analytic arguments that suggest power law distributions should be the natural null hypothesis for high variability phenomena [27].

Power law statistics are by no means unique or unexpected for wildfires. However, the impact of power law distributions for policy and decision making has not been investigated in much detail, yet is critically important, especially in the case of natural disasters. This heavy tailed statistical form is intrinsically linked with uncertainty and high variability in observations: most fires are small, while most of the area burned is contained in the few largest events. In fact, in the context of wildfires, it has been noted that the top 1% of large fires burn 80–96% of the total area [28]. Another consequence of this mathematical form is that a large event, many times bigger than anything seen recently, is not only possible but is also consistent with the distribution. This is particularly significant when planning for a worst case scenario.

Although power law statistics are observed in many systems, a particularly interesting feature is that the exponent for wildfires, 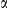, clusters around 1/2 for data sets spanning a wide range of geographic regions and time frames [4]. In general, different disaster types (e.g., fires, hurricanes, earthquakes) exhibit varying values of the exponent, suggesting that the relative scaling of physical processes, tradeoffs, and constraints may be different in each case [29]–[31]. The consistent value of the exponent 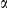 for wildfire distributions, despite varying vegetation, weather, and terrain, suggests that there may be a common mechanism that drives wildfires across a wide range of fire regimes.

Several mechanisms for power laws have been discussed in the context of wildfires, and simple models have been introduced to illustrate these mechanisms. One example is Self Organized Criticality (SOC) [32], [33], which aims to explain the presence of power laws as the consequence of a dynamical system operating at a critical phase transition. Sandpile cellular automata are the canonical example of SOC [25], combining an infinitesimal rate for discrete and spatially random reloading of the system with a threshold rule for event propagation, resulting in power law distributions. Extrapolation of this basic mechanism of slow, random regrowth with fast event propagation, terminating at grid locations where the threshold criterion for propagation is not met, has made SOC a popular topic within the complex systems community.

The phase transition underlying the SOC forest fire model is a percolation transition, associated with a critical density at which nearest neighbor connectivity first extends from one side of a discrete lattice to another. The percolation transition is fundamentally statistical, describing the average behavior of an ensemble consisting of all possible configurations of occupied (by fuel) and vacant (no fuel) sites, characterized only by the fuel density. Percolation is the most widely studied model of criticality in statistical and mathematical physics because it is so simple, enabling rigorous analysis and large scale simulations [34]. In the context of wildfires, the SOC model can be loosely interpreted as a fuel driven model, because fire propagates through connected clusters of fuel occupied sites, terminating only at vacancies. However, even in a physical model of fuel driven wildfires, the biology, ecology, chemistry, and physics of fuels is more complex than what is captured by an ensemble of random configurations of occupied and vacant sites. It is therefore no surprise that the simple, mechanistically illustrative SOC forest fire model fails to describe wildfire statistics (the exponent does not agree with observations) [35]. Taken literally, the SOC model would also predict wildland vegetation densities corresponding to the critical density in percolation, which is much lower than realistic measurements for burnable terrain [36], [37], as well as fire footprints that are tenuous and fractal, as opposed to the observed compact fire shapes [38]. Thus, the system would remain poised at the critical density even immediately after a large region spanning fire.

More recently, Highly Optimized Tolerance (HOT) [35], [39] was introduced to illustrate characteristics generally expected to arise in optimized (e.g., technology), organized (e.g., ecology), or evolved (e.g., biology) systems subject to resource constraints and a spectrum of external disturbances. The surprising result is that this abstract problem definition alone also naturally leads to a mechanism for power laws. The HOT mechanism was described initially using fire spread terminology, in order to clarify the differences between HOT and SOC. The simplest, most abstract HOT wildfire model posits that power laws emerge due to tradeoffs between vegetation yield, cost of resources, and tolerance to risks, whether that tolerance is a compromise made by corporations in a managed forest, or whether it involves biological and ecological tradeoffs between growth and resilience, adapted to conditions in a particular fire prone terrestrial ecosystem. Losses are represented as “area burned,” and “resources” represent the effort to minimize average losses. This model thus does not assume that fuel availability is the only determining factor for fire size distributions, but rather that it emerges as a consequence of some organizational process that over time favors maximization of the average yield or proliferation of vegetation well-suited to the regime. Compared to SOC, HOT is much more efficient in utilization of resources. In the context of wildfires, simple HOT models produce compact two-dimensional fires, extinguishing at the one-dimensional perimeters. HOT leads to high vegetation densities, much more representative of natural wild lands, and HOT also leads to heavy tailed size distributions. This can be understood because optimizing the system for common events leads to catastrophically large rare events. Interestingly, HOT produces a power law that matches both wildfire data 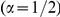 and detailed fire simulations [4], [40]–[42].

In this work we posit an alternate simple model in which we focus on temporal dynamics rather than spatial features. Our first objective is to understand the extent to which the dynamics of fire growth and suppression can be understood from models that capture the temporal features but abstract the spatial features. The second objective is to use this simplified temporal framework to assess tradeoffs that result from basic tensions in fire response policy: fire growth over time, limited resources, and decisions about when to deploy resources and how many to send. Our study takes an important preliminary step in the development of future applications that facilitate real-time response efforts in coupled human and natural systems.

## Methods

### Natural Fire Dynamics

We begin with a minimal model of fire growth and natural fire extinction, based on the physical processes known to drive fire behavior. In the next section, we augment the model to include human suppression explicitly. To capture the temporal dynamics of wildfire, we consider a finite number of discrete parcels of burnable substrate that can be affected by fire. We associate each parcel with one of three states: *unburned*, *burning*, and *burned*. Our simulations start with some number of burning parcels and the rest of the parcels in the unburned state. An unburned parcel can catch fire to enter the burning state and will eventually die out, becoming burned. The time scale under consideration here does not allow for regrowth of a burned parcel, so these state transitions proceed in one direction only.

We refer to burning parcels as *firelets* and assume that they can spread to unburned parcels, i.e. firelets can give “birth” to additional firelets. Although the notion of discrete parcels is geospatially motivated, our rules for fire spread ignore spatial features. However, spatial scale is implicit in our model in two important ways. First, the rate at which a firelet gives birth to other firelets assumes an underlying length scale associated with the size of the parcel and the rate at which fire spreads across it. Second, the total number of parcels *J*, which we refer to loosely as the *size of the burnable region*, sets an upper bound 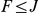 on fire footprint *F* represented in our model. Note that *J* need not be interpreted literally as the total area of a particular region, but instead may be more directly linked to some user specified threshold area, perhaps associated with a scale at which the fire exceeds the capacity of locally available suppression resources, and is deemed *out of control.* For this interpretation, a real wildfire may continue to expand, consuming an even larger area, but growth beyond this size is not explicitly represented numerically.

A finite population of parcels *J* constrains the behavior of the model in two ways. First, we have an upper bound on the number of potential firelets. As the number of unburned parcels decreases over time, the upper bound on firelets also decreases. Second, all fires are finite in duration and ultimately end with the fire going out (a trapping state), because the fire has consumed the entire burnable region 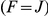 or because the fire stopped short of burning everything 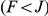. In either case, system behavior is transient, so steady state calculations of fire behavior are not meaningful. Instead, we examine the effects of model parameters on the number of burned parcels *F*.

We model the transitions for a population of parcels as state-dependent and stochastic. Let *j* denote the current number of firelets. Let 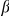 be the constant rate at which a single firelet gives rise (or *birth*) to a new firelet. Let 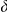 be the constant rate at which a single firelet is extinguished (or *dies*) on its own (i.e., in the absence of human suppression). Then, the state transitions of the population as a whole can be understood in terms of a birth-death process where in state *j* the aggregate birth rate is 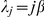 and the aggregate death rate is 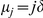. We choose the simplest assumptions: all burning firelets are actively spreading and can also die at any time. To simulate continuous time dynamics we model the time interval until the next birth or death as an exponentially distributed random variable, *t*. The probability density function for *t* is: 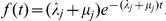 This implies that the existence of more firelets increases the probability that in any fixed time interval at least one of them either spreads or dies out. The specific choice of rates underlies quantitative relationships (on average) between duration and size *F* and the characteristic time scale associated with system-wide events.

### Time Series of Individual Fires

Fire behavior is driven primarily by the ratio of the state-dependent firelet arrival 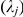 and departure 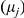 rates. When 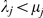 the fire tends to decrease in size, and when 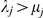 the fire tends to increase in size. Here by construction, the ratio 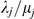 is constant for all values of *j* and characterized by the scaled growth rate 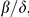 which serves as a proxy for physical conditions such as weather, vegetation moisture content, and atmospheric humidity. For example, high winds may be represented by an increase in the birth rate 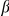, and a higher scaled growth rate. Similarly, high vegetation moisture may be represented by an increase in the natural death rate 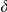, and a lower scaled growth rate. For simplicity we drop the term ‘scaled’ and refer to 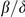 as the *growth rate*.

In the analysis to follow, we select growth rate values that yield size distributions roughly matching size distribution data. In principle, the growth rate itself could be estimated for a given set of environmental factors, by combining empirical measurements with a theoretical understanding of fire propagation. For example, the Rothermel equation [43], [44] takes wind, vegetation, and terrain as inputs to estimate the fire spread rate, which in our case corresponds to 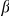. Fuel composition, and therefore the energy released via combustion, would determine the rate 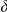 at which the fire ceases to be actively spreading.

**Figure 1:**
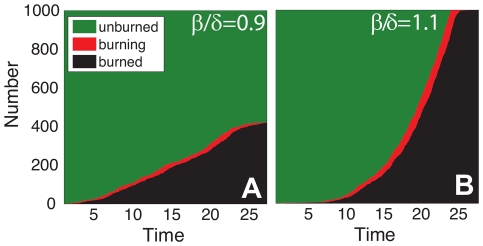
Individual fire simulations in the absence of suppression for (A) mild, and (B) extreme conditions. The total number of parcels 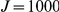 is divided between unburned (green), burning (red), and burned (black) according to the stochastic dynamics of the birth-death process. The fire terminates when the number of burning firelets *j* first reaches zero. In (A) the final fire size *F* is roughly 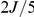, while in (B) 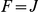.

Figure 1 illustrates trajectories for two simulated fires. At each discrete time step we display the number of parcels that are in the burning (i.e., the *j* firelets), burned, and unburned states. As one would expect, *j* fluctuates over time, with the fire ending when 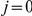. Because the total population *J* is constant and finite, the number of burned parcels is nondecreasing in time while the number of unburned parcels is nonincreasing.

Figure 1A shows a simulated fire with growth rate 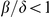, a condition we refer to as *mild*. This fire dies out with a nonzero number of unburned parcels remaining 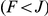, which is typical for mild conditions. Figure 1B shows a fire with growth rate 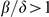, which we refer to as *extreme* conditions. The simulated fire continues until there are no remaining unburned parcels 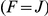. Once this size limit is reached, the number of firelets steadily decreases until the fire is out. Fires that grow *out of control* and span the system are common in extreme conditions. In both cases, the number of firelets at any point in time is only a small fraction of the total number of burned parcels, of order 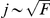, consistent with the perimeter of a compact, two-dimensional fire footprint.

## Results

### Fire Size Distributions

**Figure 2:**
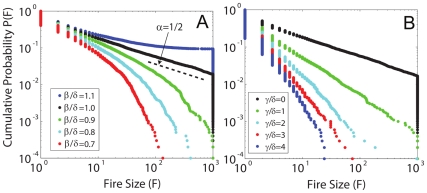
Fire size distributions 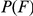 calculated for varying growth and suppression rates. Results are based on 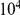 simulation runs for a fire in a region containing burnable substrate of 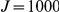 parcels. In (A), growth rate 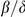 varies in the absence of suppression. Dangerous conditions are described by a pure power law with exponent 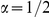. Extreme conditions 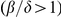 lead to excess weight in the tail, which can be interpreted as fires that have surpassed a threshold value and can no longer be controlled. Finally, mild conditions 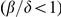 result in a sharp (exponential) cutoff 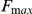, which decreases as 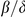 decreases. In (B), the suppression rate 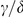 varies in dangerous conditions 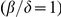. Pure power laws are obtained for a range of 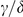, with an increasing exponent (steepening slopes) as suppression is increased.

Size statistics 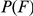, obtained by simulating catalogs of 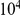 individual fires, are illustrated for different growth rates in Figure 2A. For mild conditions, fires generally go out before consuming the entire burnable region (total number of parcels 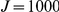), and 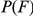 can be approximated by a power law with a large scale cutoff characterized by some 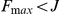. In contrast, for extreme conditions fires are generally much larger and there is an accumulation of top ranked fires that burn all 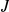 parcels. This value can be understood as a threshold beyond which the fires cannot be controlled.

Interestingly, for 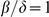 (a case we denote as *dangerous* conditions), 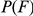 is an exact power law with exponent 1/2, similar to observed wildfire statistics [4]. Mathematically, the origin of this exponent can be understood in terms of a discrete random walk in which each “step” corresponds to a state transition in the birth-death process. Our 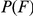 maps directly onto the distribution of *first return times* (the number of firelets corresponds to displacement of the random walker, and fire size maps to time), which is a power law with exponent 1/2. For a mathematical proof, see [45].

To what extent does our model capture some basic features of fire spread even without explicit spatial rules? If we think of the number of firelets as the region of the fire that is actively spreading, then the number of firelets corresponds to the perimeter or “front” of the fire. If the birth rate and death rate in our model are exactly balanced, each firelet will on average spread to one other firelet before dying out. This is consistent with spread governed by a quasi-linear, one-dimensional, perimeter of a compact, two-dimensional fire shape. Thus, a growth rate 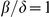 may be a particularly useful choice of parameters to capture key features of fire spread.

In this analysis we have explored a relatively narrow range of growth rates around 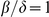, since this is the regime that most closely resembles wildfire data. Significantly higher values result in a monotonically larger fraction of fires burning all parcels while much lower values result in only small events. Neither of these extremes captures the relevant features seen in wildfire data: an abundance of small events, and a heavy tail comprised of rare, large events. However, if 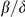 is assumed to vary in time, the variance of this parameter can be expanded without necessarily creating anomalous distributions. This will be explored in future work.

### Coupled Fire and Suppression Dynamics

We include human suppression in the dynamics of the fire by adding a decision variable 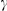, representing the total resources assigned to the fire. This modifies the death rate for a system with *j* firelets, so that it depends on both the natural extinction rate per firelet, 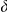, and the suppression rate, 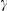: 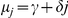. Note that the behavior of this model can be fully characterized by the initial size of the fire (*s*), the scaled growth rate 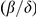, and the scaled suppression rate 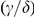. The scaled suppression rate captures the relationship between suppression due to resources allocated and the natural extinction rate. As before, we drop the specification ‘scaled’ and simply refer to the *suppression rate*. As with observed wildfires, a larger fire requires more suppression resources.

### Tradeoff One: Initial Size versus Suppression Rate

Figure 2B illustrates 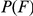 for dangerous conditions and varying suppression. When 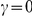, the simulation produces a pure power law with exponent 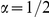 (Figure 2A). In comparison, increasing suppression results in monotonically smaller fires. For all of the illustrated values of 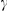, the statistics remain well described by power laws, extending to large event sizes, although the exponents increase (the curves become steeper) as 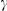 increases. This implies that the probability of a large, system spanning event remains non-negligible. In contrast, decreasing the natural growth rate in Figure 2A results in progressively smaller exponential cutoffs in the tail of 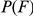, so the probability of extremely large fires becomes increasingly remote. Thus, varying the suppression rate does not affect the largest events as significantly as varying the growth rate, reflecting the dominance of environmental factors over a fixed suppression allocation as the number of firelets becomes large.

The distributions in Figure 2A and 2B are for simulated fires initiated with 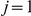. Here, the implicit assumption is that the delay for the arrival of suppression resources is negligible. In more realistic situations, the fire is likely to have grown to some larger size by the time suppression resources arrive, and this “initial” size (i.e., at the time of arrival) may determine whether or not the fire is contained. In what follows, we vary the initial size as a proxy for fire growth during this delay, starting the simulations with 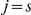 firelets and with 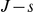 unburned parcels.

**Figure 3:**
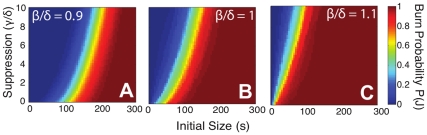
Burn probabilities 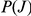 as a function of initial size *s* and suppression rate 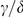. Conditions are mild in (A), dangerous in (B), and extreme in (C). As the conditions become increasingly severe, the transition from low to high 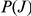 shifts to smaller initial sizes. Results shown represent averages for 100 fires.

We define the *burn probability* 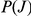 to be the probability that the fire consumes the entire burnable region, and investigate 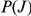 as a function of the initial size (*s*), growth rate 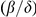, and suppression rate 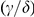. Figure 3 illustrates our results obtained by simulating 100 fires for each set of parameters. Panels A–C in Figure 3 compare the results for mild, dangerous, and extreme conditions. In each case, the larger the initial size, the greater the suppression rate required to contain it. Comparing the results for different conditions shows that each 10% increase in the growth rate requires roughly an order of magnitude decrease in initial size in order for the same suppression rate to be effective in keeping the burn probability low.

**Figure 4:**
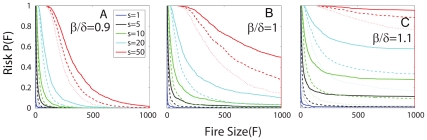
Risk curves 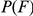 as a function of initial size *s* and suppression rate. Conditions are mild in (A), dangerous in (B), and extreme in (C). In each case the line style indicates suppression rate: 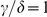 (solid), 2 (dashed), and 3 (dotted). Burn probability 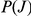 corresponds to 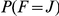, with 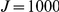. In mild conditions risk generally remains low, unless the initial size is large. In dangerous conditions suppression plays a significant role in reducing risk across the full range of initial sizes. In extreme conditions suppression is most effective when the initial size is small. Beyond a certain size, the fire is increasingly likely to grow out of control.

Risk curves [46] generalize the concept of burn probability 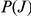 to include the full range of sizes *F*. They are essential ingredients for any interactive, dynamic decision making tool, which might be used to estimate the suppression rate required to maintain the fire at a certain risk level based on the current conditions and the size of the fire. Here 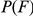 corresponds to the *risk* that the fire will reach size *F* or greater. Figure 4 illustrates the results under mild, dangerous, and extreme conditions. As expected, larger initial sizes *s* result in increased risk of a large fire. As the suppression rate increases (going from solid to dashed to dotted lines), the risk of large fires decreases. In mild conditions the initial size plays the dominant role in determining 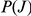, and the suppression rate has relatively little impact. In dangerous conditions the suppression rate has a much more significant effect, especially for fires with a larger initial size. Finally, in extreme conditions the risk curves flatten out, reflecting the likelihood of a size 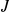 event for a wide range of initial sizes and suppression rates.

### Tradeoff Two: Time Delay versus Suppression Level

In practice, the decision maker will not know in advance the size of the fire when suppression resources arrive. This size will depend on the initial size of the fire when discovered, prevailing environmental conditions, and the length of time that the fire grows before resources arrive. Typically, a longer time delay results in a larger fire and a need for more resources to maintain a low burn probability.

We evaluate the tradeoff between the time delay and resource requirements by adding an explicit time dependence 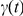. For simplicity, we take 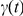 to be a threshold function in time. Letting 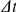 denote the time delay, we have 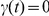 for 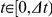, and 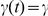 for 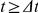. The function 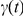 could be made more complicated, to model different resources arriving from different locations at different times. However, even this minimal form is consistent with a simple response policy: once a fire is detected, send an initial response force of some standard size. In general, the arrival time will depend on the distance between the fire and the fire station and the difficulty of traversing the terrain.

**Figure 5:**
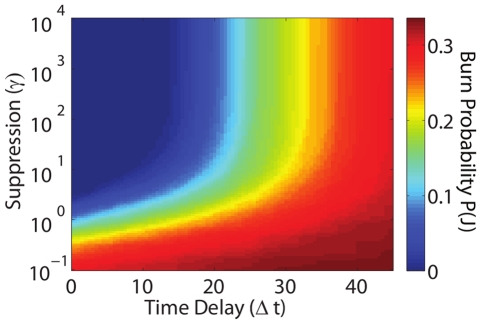
Tradeoffs between time delay 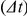 and suppression rate 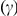 represented by contours of constant burn probability 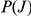. At small time delays, 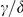 must exceed some minimum value to keep 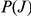 low. As 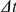 increases, initially suppression rate must increase roughly exponentially with delay to maintain a constant 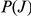. At a larger value of 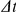, there is a sharp transition where the contours become essentially vertical, corresponding to a delay, determined by the underlying rates and system size, beyond which the fire is likely to have either burned out or grow out of control. Results shown represent dangerous conditions 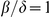 and averages over 1000 simulations and all fires are initialized with size 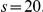

Figure 5 shows the tradeoffs between time delay and suppression rate; we plot burn probability as a function of 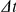 and 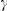, under dangerous conditions. Even for instantaneous arrival 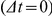 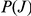 is non-negligible for small values of the suppression rate (roughly 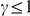), suggesting that an initial response, no matter how prompt, may be ineffective if it is too small. For small values of 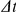, contours of constant 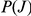 are roughly linear in semi-log space: 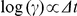. This indicates that the suppression strength 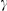 must increase on average exponentially with the delay 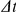 to keep pace with the growing fire.

Beyond a characteristic time, the slope of the constant 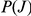 contours increases sharply with 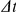, and 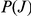 becomes independent of the suppression level. The system enters this regime at approximately a suppression rate of 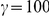. This suppression level roughly corresponds to 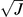, the entire actively burning region (or “perimeter”) of a fire that burns all parcels. Hence, at this point the suppression effort either reaches the fire in time and is extremely effective, or the fire has already consumed the entire burnable region and suppression has no effect.

### Tradeoff Three: Resource Division for Two Simultaneous Fires

Dynamic decisions involving resources are especially critical when considering two or more wildfires at different locations, spreading at different rates. In many situations a portion of resources may be directed to a new outbreak, even if they are currently in use elsewhere on another, possibly larger, fire. The rationale is that early response is generally a priority because it increases the likelihood of containment.

To investigate tradeoffs involving the division of resources, we consider two fires (denoted A and B). We implicitly incorporate the notion of delays in ignition and response in the initial sizes of the two fires, 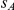 and 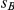, taking 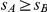, so that Fire *B* may be thought of as the *new* fire. We assume the conditions for Fire A are dangerous, and we vary the conditions for Fire B from mild to extreme. We place a cap of 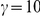 on the total resources available, to be divided between the two fires: 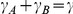. Without loss of generality, we set 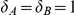. We seek the optimal division of suppression resources between these fires, where optimal is determined by minimizing the combined size of the two fires 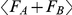 (i.e., as a proxy for damage), averaged over 1000 runs.

**Figure 6:**
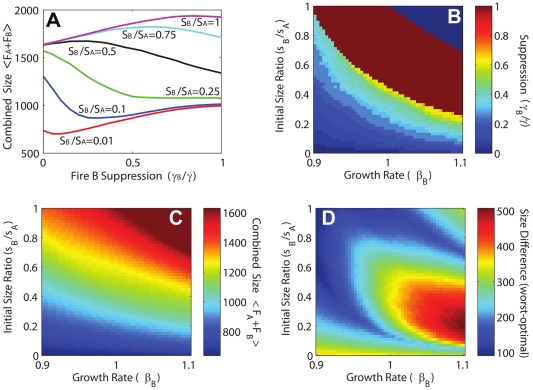
Resource optimization for two simultaneous fires, Fire A and Fire B, subject to the constraint 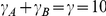. Fire A has dangerous conditions and initial size 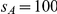. In panel (A) the optimization procedure is shown explicitly for 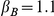 (extreme case), and varying values of 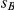. Optimal solutions correspond to the minimum mean total size 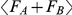 for each curve, and begin with a small allocation to Fire B when 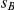 is small, rapidly shifting resources to Fire B as 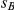 increases, and then shifting resources back to Fire A when 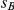 becomes sufficiently large. Results represent averages over 1000 realizations. In panel (B) these results are extended to values of 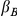 extending from mild to extreme conditions. The trends in panel (A) persist for all cases where Fire B is extreme. When Fire B has dangerous or mild conditions, resources are not shifted back to Fire A when 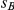 becomes large. Panel (C) illustrates the value of 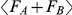 associated with the optimal solution in panel (B). Here 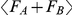 serves as a proxy for total damage. Figure (D) shows the size difference between solutions corresponding to the maximum (worst case) and minimum (optimal) value of 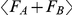. Regions where the difference is elevated correspond to regimes where optimal decision making has the most significant impact.

Figure 6 summarizes our results. Panel (A) explicitly illustrates the optimization process when the conditions for Fire B are extreme. The growth rate in panel (A) corresponds to the limiting case (right most boundary) shown in the remaining panels, representing the optimal resource division (B), the total burn area of the pair of fires (C), and the difference in burn area between the optimal solution and the worst case scenario (D).

For each curve in Figure 6A, optimization identifies the fractional resource allocation 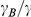 to Fire B that minimizes the mean total size. When 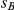 is small, the optimal solution is a small allocation to Fire B, but as 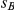 increases the optimal allocation to Fire B increases rapidly and then shifts completely back to Fire A as 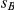 becomes comparable to 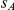. In that case, because Fire B is spreading more rapidly, there is a greater advantage in concentrating suppression resources on Fire A.

Figure 6B shows that this general pattern persists while the conditions for Fire B remain extreme. The contours shift upwards as the severity of the conditions for Fire B decreases, implying comparable resource assignments become optimal at larger initial sizes of Fire B. Interestingly, the final transition from full allocation to Fire B, back to full allocation to Fire A is extremely abrupt (represented by the discrete color shift in the upper right corner of Figure 6B), corresponding to a discontinuous transition from a solution that employs all available resources to combat the smaller, more rapidly growing Fire B, to a solution that gives up on Fire B, and uses all the resources for Fire A. When the conditions for Fire B are dangerous or mild 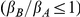, the solutions are similar, although in that case it is never advantageous to move resources back to Fire A as 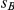 increases, because now 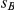 is the more slowly growing fire, so when the sizes become comparable, resources are most effective for Fire B.

Figure 6C illustrates the combined size 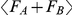 corresponding to the optimal resource divisions computed in 6B. Here values are strictly bounded below by the sum of the initial sizes of the two fires 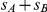 (recall 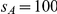, and 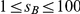), and bounded above by 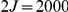. The transition from small (lower left) to large (upper right) combined fire size is a relatively smooth crossover, unlike the optimal resource allocation contours in Figure 6B, which exhibit sharp crossovers and abrupt transitions. In the case of two large fires, the initial size and growth rate of Fire B is sufficiently large that the burn probability for Fire B approaches unity. In this case, the size of Fire B is typically near J, and the resources are concentrated on suppressing Fire A, which on average is contained at a size near 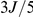.

To estimate the impact of optimal decision making, we compare the combined size of the optimal solution in Figure 6C to the size that corresponds to assigning resources in a manner that *maximizes* the combined size of the two fires (this corresponds to choosing the value of 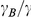 in Figure 6A that maximizes, rather than minimizes, mean total size for each curve). This *worst case scenario* enables us to identify regimes in which optimization leads to the most significant gains. Figure 6D illustrates the difference between the maximum and minimum combined size. Interestingly, the net gain obtained through optimization exhibits three distinct regions with elevated values. One occurs along the lower boundary, where the optimal solution allocates only minimal resources to Fire B, while the worst case solution corresponds to assigning most of the resources to Fire B. Another peak occurs in the upper right corner, where the optimal solution corresponds to allocating all the resources to Fire A, because Fire B is sufficiently large and rapidly growing so that the resources have minimal impact and the burn probability is essentially equal to one. Here the worst case solution leaves all the resources with Fire B, so that in the end, both fires, rather than just one, tend to burn to size 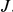 The third, largest and broadest peak in Figure 6D occurs near the lower right corner, corresponding to a small initial size and rapid growth rate for Fire B. It is in this region where judicious allocation of resources has the greatest impact on keeping both fires under control.

## Discussion

Recent natural disasters worldwide highlight the urgent need for real-time, dynamic, decision making tools for response [47]. In the past, there has been extensive work developing hazard maps, and other static tools that chart current conditions and estimate the probability of regional hazards for policy makers and responders [48]–[50]. In contrast, the next generation of dynamic tools will have the capacity to evolve in real time with both the threat and the response, and use probabilistic methods to estimate the outcome based on the combined disaster and decision making trajectories.

In principle, the information fed into these tools could be as detailed as desired and involve a combination of observations and modeling. In the case of fire, it could combine physical models of fire spread and atmospheric conditions, with high resolution measurements of topography, fuel conditions, and critical infrastructure [51]–[53]. For response, it could include the mobility, potency, and cost of different platforms–aircraft, helicopters, bulldozers, or engines. Each of these inputs has seen significant advances in recent years–increases in computational capacity and satellite observations have improved the resolution of models and observations, and new technologies for firefighting and detection have improved the ability to sense and respond.

The work presented in this paper begins to address what we perceive to be a critical missing link in these recent advances–development of a robust framework for combining and distilling dynamic information in a manner that is useful for policy and decision making. Although we have focused on wildfire response in this paper, we believe that the need for this connection exists more broadly in other situations involving coupled dynamics of disaster and response. The ultimate end-product of this work might be a tool that would enable information from a variety of sources to be summarized in an evolving decision diagram with cost projections carried forward over a user-designated time interval. For example, such a diagram might summarize model and observation based projections, constrained by resource availability and effectiveness, while the user considers deployment in different locations at specific times. In the context of the work presented here, our input parameters: the natural growth 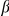 and extinction 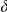 rates, the initial size *s*, and the suppression allocation 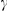 represent real-time inputs based on current conditions, models, and policy decisions, with dynamic outputs, analogous to our probability contours, highlighting the essential tradeoffs and estimating risks over various time scales.

Of course, a great deal must be done before such complex analysis can be automated. Current capabilities to observe and model greatly exceed the capacity to distill and integrate results in a manner that is comprehensible, efficient, and robust for policy. Any effort to compress information is inherently delicate because of the intrinsic fragilities associated with cascading breakdowns initiated by one small failure. This motivates our approach, based on deliberately transparent models and isolated tradeoffs, aimed at clarifying fundamental issues that arise generally in this class of problems.

In these first steps of developing a decision making framework for policy, it is less crucial to capture every detail of wildfire dynamics than it is to correctly represent those features that are resolved in the initial, coarse representation that is used–in this case spread rates consistent with compact fire footprints and the resulting statistical distribution of sizes. Such features are expected to persist as more detailed models are incorporated into the framework. In contrast, a framework based on a model that does not get the simple things right is likely to produce misleading results from the start. More detailed models of fire spread retain compact shapes and statistical features, while incorporating additional features (e.g., topography and fuel type), and exposing additional sensitivities (e.g., winds and humidity) that are important for fire spread.

This work suggests many directions for future research–integration with geospatial approaches, increases in fire and response model resolution, and more accurate, data-driven incorporation of physical parameters. In the case of California wildfires, a dominant factor is wind. The largest, most destructive wildfires are typically associated with extreme, high wind, low humidity Santa Ana conditions [54], [55]. Fluctuations in wind speeds during these events dominate both the dynamics of fire spread and the effectiveness of response. Even in our abstract model, this could be represented by time dependent birth and death rates for fire spread, to evaluate the optimal time dependence for effective response.

Current decision making for wildfire relies on a combination of protocols established in advance and real-time expert opinion. A great deal of data is available to facilitate these decisions. In some cases, it may be even too much, resulting in information overload. The purpose of investigations such as ours is not to replace the experts, but rather to provide them with a sound, statistical basis for synthesizing information, establishing protocols, and predicting system sensitivities in advance.

## References

[1] Perry GLW (1998). Current approaches to modelling the spread of wildland fire: A review.. Progress in Physical Geography.

[2] Weber RO (1991). Modelling fire spread through fuel beds.. Progress in Energy and Combustion Science.

[3] Barbour MG, Burk JH, Pitts WD (1987). Terrestrial plant ecology, 2nd edition..

[4] Moritz MA, Morais ME, Summerell LA, Carlson JM, Doyle J (2005). Wildfires, complexity, and highly optimized tolerance.. Proceedings of the National Academy of Sciences of the United States of America.

[5] California Department of Forestry and Fire Protection: Statistics and events.. http://cdfdata.fire.ca.gov/incidents/incidents.

[6] Big burn: The Times explores the growth and cost of wildfires.. http://www.latimes.com/news/local/la-me-fire-index,0,4857752.htmlstory.

[7] Gebert KM, Calkin DE, Yoder J (2007). Estimating suppression expenditures for individual large wildland fires.. Western Journal of Applied Forestry.

[8] Calkin DE, Gebert KM, Jones JG, Neilson RP (2005). Forest service large fire area burned and suppression expenditure trends, 1970–2002.. Journal of Forestry.

[9] Westerling AL, Hidalgo HG, Cayan DR, Swetnam TW (2006). Warming and earlier spring increase western U.S. forest wildfire activity.. Science.

[10] Westerling AL, Gershunov A, Brown TJ, Cayan DR, Dettinger MD (2003). Climate and wildfire in the western United States.. Bulletin of the American Meteorological Society.

[11] Swetnam TW, Betancourt JL (1998). Mesoscale disturbance and ecological response to decadal climatic variability in the American Southwest.. Journal of Climate.

[12] Snyder GW (1999). Strategic holistic integrated planning for the future: Fire protection in the urban/rural/wildland interface (URWIN)..

[13] Radeloff VC, Hammer RB, Stewart SI, Fried JS, Holcomb SS (2005). The wildland-urban interface in the United States.. Ecological Applications.

[14] Islam KMS, Martell DL (1998). Performance of initial attack airtanker systems with interacting bases and variable initial attack ranges.. Canadian Journal of Forest Research.

[15] Bookbinder JH, Martell DL (1979). Time-dependent queueing approach to helicopter allocation for forest fire initial-attack.. INFOR: Information Systems and Operational Research.

[16] Martin-Fernández S, Martínez-Falero E, Pérez-González JM (2002). Optimization of the resources management in fighting wildfires.. Environmental Management.

[17] Donovan GH, Rideout DB (2003). An integer programming model to optimize resource allocation for wildfire containment.. Forest Science.

[18] Martell DL (1982). A review of operational research studies in forest fire management.. Canadian Journal of Forest Research.

[19] Haight RG, Fried JS (2007). Deploying wildland fire suppression resources with a scenario-based standard response model.. INFOR: Information Systems and Operational Research.

[20] Pisarenko V, Rodkin M (2010). Heavy-tailed distributions in disaster analysis..

[21] Hergarten S (2004). Aspects of risk assessment in power-law distributed natural hazards.. Natural Hazards and Earth System Science.

[22] Mitzenmacher M (2004). A brief history of generative models for power law and lognormal distributions.. Internet Mathematics.

[23] Sornette D (2006). Critical phenomena in natural sciences, 2nd edition..

[24] Newman MEJ (2010). Networks: An introduction..

[25] Bak P (1996). How nature works: The science of self-organized criticality..

[26] Barabási AL (2002). Linked: The new science of networks..

[27] Willinger W, Alderson DA, Doyle JC, Li L (2004). More “normal” than normal: Scaling distributions and complex systems.. Ingalls RG, Rossetti MD, Smith JS, Peters BA, editors, Proceedings of the 2004 Winter Simulation Conference, New York: ACM Press.

[28] Strauss D, Bednar L, Mees R (1989). Do one percent of forest fires cause ninety-nine percent of the damage?. Forest Science.

[29] Malamud BD, Morein G, Turcotte DL (1998). Forest fires: An example of self-organized critical behavior.. Science.

[30] Corral A, Ossó A, Llebot J (2010). Scaling of tropical-cyclone dissipation.. Nature Physics.

[31] Gutenberg B, Richter CF (1954). Seismicity of the earth and associated phenomena, 2nd edition..

[32] Chen K, Bak P, Jensen MH (1990). A deterministic critical forest fire model.. Physics Letters A.

[33] Drossel B, Schwabl F (1992). Self-organized critical forest-fire model.. Physical Review Letters.

[34] Stauffer D, Aharony A (1992). Introduction to percolation theory, 2nd edition..

[35] Carlson JM, Doyle J (2000). Power laws, highly optimized tolerance, and generalized source coding.. Physical Review Letters.

[36] Christensen GA, Campbell SJ, Fried JS (2008). California’s forest resources, 2001–2005: Five-year forest inventory and analysis report..

[37] California Department of Forestry and Fire Protection: Surface fuels maps and data.. http://frap.cdf.ca.gov/data/fire.

[38] Anderson HE (1983). Predicting wind-driven wild land fire size and shape..

[39] Carlson JM, Doyle J (1999). Highly optimized tolerance: A mechanism for power laws in designed systems.. Physical Review E.

[40] Carlson JM, Doyle J (2002). Complexity and robustness.. Proceedings of the National Academy of Sciences of the United States of America.

[41] Peterson SH, Morais ME, Carlson JM, Dennison PE, Roberts DA (2009). Using HFire for spatial modeling of fire in shrublands..

[42] Peterson SH, Moritz MA, Morais ME, Dennison PE, Carlson JM (2011). Modelling long-term fire regimes of southern California shrublands.. International Journal of Wildland Fire.

[43] Rothermel RC (1972). A mathematical model for predicting fire spread in wildland fuels..

[44] Rothermel RC (1983). How to predict the spread and intensity of forest and range fires..

[45] Newman MEJ (2005). Power laws, Pareto distributions and Zipf’s law.. Contemporary Physics.

[46] Kaplan S, Garrick BJ (1981). On the quantitative definition of risk.. Risk Analysis.

[47] Centre for Research on the Epidemiology of Disasters: 2010 disasters in numbers.. http://cred.be/sites/default/files/PressConference2010.pdf.

[48] United States Geological Survey hazard mapping images and data.. http://earthquake.usgs.gov/hazards/products/.

[49] California Department of Forestry and Fire Protection: Fire hazard maps.. http://frap.cdf.ca.gov/data/frapgismaps/select.asp?theme=5.

[50] National Oceanic and Atmospheric Administration: National weather hazards map.. http://www.weather.gov/largemap.php.

[51] Finney MA, Grenfell IC, McHugh CW, Seli RC, Trethewey D (2011). A method for ensemble wildland fire simulation.. Environmental Modeling and Assessment.

[52] Calkin DE, Rieck JD, Hyde KD, Kaiden JD (2011). Built structure identification in wildland fire decision support.. International Journal of Wildland Fire.

[53] Calkin DE, Thompson MP, Finney MA, Hyde KD (2011). A real-time risk assessment tool supporting wildland fire decisionmaking.. Journal of Forestry.

[54] Moritz MA (1997). Analyzing extreme disturbance events: Fire in Los Padres National Forest.. Ecological Applications.

[55] Davis FW, Michaelsen J (1995). Sensitivity of fire regime in chaparral ecosystems to climate change.. Moreno JM, OechelWC, editors, Global change and Mediterranean-type ecosystems, New York: Springer.

